# Lobaplatin Inhibits the Proliferation of Hepatollular Carcinoma Through P53 Apoptosis Axis

**DOI:** 10.5812/hepatmon.6024

**Published:** 2012-10-11

**Authors:** Ying Wang, Wen-Ling Zheng, Wen-Li Ma

**Affiliations:** 1 Institute of Genetics Engineering, South Medical University, Tonghe, Guangzhou, China

**Keywords:** Lobaplatin, Carcinoma, Hepatocellular, E2F1

Cancer is a major killer nowadays, just as its name-a kind of hideous arthropods- which always goes through everywhere without restraints. little is known about how to repress its proliferation and destroy its defense. A lot of researchers focus on the antineoplastic agents which may suppress the proliferation and metastasis of Hepatocellular carcinoma(HCC) (1). HCC is the sixth most common tumor and the third leading cause of cancer deaths worldwide, particularly in Eastern Asia (2). Every year, billions of money is put into the researches about new drug account for HCC and the mechanism on apoptosis and cell cycle arrest of hepatoma cells (1, 3) Then the third generation platinum compounds emerge in time for the demand. Lobaplatin (D-19466;1,2- diammino-methyl-cyclobutaneplatium (II)-lactate) is one of platinum compounds and has presented encouraged anti-carcinoma activity in HCC without significant hepatotoxicity (4).

The discovery of platinum was serendipitous. One day, Rosenberg observed that the division of bacteria ceased and their size increased about 300-fold and something was different from before was the platinum-conducting plate, then he deduced that the platinum species cause the inhibition of bacteria division (5). Later he applied cis-platin on tumor cells to see if platinum could also suppress tumor cells’ growth and at last his Positive conjecture was proven.The discovery of Rosenberg accelerated the researches of platinum species in clinical trials as a kind of novel antitumor drugs;the first generation platin-based drugs. For various kinds of cancers, such as ovarian cancer, bladder cancer, colorectal cancer and etc., platinum indeed gained lots of success with a few side effects (6-8). Recently, researchers have developed the third generation derivative , oxaliplatin and lobaplatin which were more active and tolerable against cancer in combination with other drugs (9, 10). In the case of HCC and other tumors, lobaplatin could inhibit cell proliferation effectively and overcome some forms of resistance to cisplatin in preclinical tumor models as a new platinum drug (11, 12). Lobaplatin inhibited the proliferation of human HCC cells mostly by activating apoptosis and cell cycle arrest. according to Wu’s research, they found lobaplatin that could repress the proliferation of 5 HCC cell lines by MTT proliferation assay, with IC50 values ranging from 1.45 to 5.22μg/ml.The most sensitive cell line was P53 wild-type SMMC-7721,and the others were p53 null Hep 3B, p53 mutant Huh-7.suggesting in response to lobaplatin, p53 was active in cell signaling cascade. The latter experiment has proved it:by p53 WT SMMC-7721 they found an sub-G1 apoptotic peak but in p53 null Hep 3B none was found (13). A common truth was that chronic hepatitis B virus(HBV) infection contributed dominantly to HCC, and HBx suppressed the p53 induced apoptosis to promote HCC progression (14). HBx-p53 reciprocal transcription repression was so important that p53 related pathways were key targets in HCC drugs development (15). furthermore Wu et al. assessed the regulation of p53 expression in lobaplatin signaling pathway.They demonstrated that p53 upregulation was related to E2F1/Rb in lobaplatin treated HCC cells by RT-PCR and western blotting methods. As we all know,E2F1 is a member of E2F transcription factors family which plays an increasingly-appreciated role in regulating cell-cycle progression and programmed cell death (16). E2F1 and p53 formed an apoptotic axis to control the cascade in response to DNA damage (17, 18). according to Choi M’s report, E2F1 could overcome the effect of HBx on p53 promoter and it activated p53 promoter though the E2F1 binding site (19).gathering this and the relationship of p53 and E2F1/Rb in lobaplatin treatment, we could conclude that lobaplatin is involved in p53 apoptosis axis though its Proliferation inhibitance and progression of HCC. Elucidation of the function mechanism of the novel platin-based drug might be helpful for new molecular targets’ researches and the development of chemotherapy. Another interesting aspect of their wok was a time-dependent cell cycle arrest in G0/G1 and G2/M phase in response to lobaplatin. Flow cytometry analysis revealed that with incubation of lobaplatin, SMMC-7721 cells were arrested in G1 phase continuously 24hr after treatment and differences in p53-null cell lines were not significant (13).The upregulation of p53 and its necessary role in lobaplatin therapy is very promising and may develop new avenues for HCC therapeutic intervention.

Although researchers assumed that lobaplatin helps to activate the innate defense responses to tumors, one should not disregard the resistance is still exist, although it was much less than that to cisplatin (20). Up to now, about 35 platin-based drugs are entered clinical trials for the screen on the candidate with at least side-effects and resistances (21). it was the motivation for tremendous efforts in the disclosure of the underline signaling pathway for the functional mechanism and resistance of the drug. In conclude, we have a so long way to go, which is burdensome but promising.
